# Association Between Net Vertebral Artery Flow Volume and Non-AF Stroke: A Retrospective 2-Year Analysis

**DOI:** 10.3389/fneur.2018.01198

**Published:** 2019-01-18

**Authors:** Hsun-Hua Lee, Li-Kai Huang, Hwai-Jan Chang, Dean Wu, Nai-Fang Chi, Lung Chan, Chaur-Jong Hu, Chih-Chung Chen

**Affiliations:** ^1^Department of Neurology, Taipei Medical University–Shuang Ho Hospital, New Taipei City, Taiwan; ^2^Dizziness and Balance Disorder Center, Taipei Medical University–Shuang Ho Hospital, New Taipei City, Taiwan; ^3^Taipei Neuroscience Institute, Taipei Medical University, New Taipei City, Taiwan; ^4^Department of Neurology, School of Medicine, College of Medicine, Taipei Medical University, Taipei, Taiwan; ^5^Stroke Center, Taipei Medical University–Shuang Ho Hospital, New Taipei City, Taiwan

**Keywords:** anterior circulation infarction, DWI, flow volume, posterior circulation infarction, sonography, stroke subtypes

## Abstract

**Objectives:** Association between net vertebral artery flow volume (NVAFV) and stroke types remains unclear. We hypothesize NVAFV is low in patients with posterior circulation infarction (PCI) and an ideal cut-off value for discriminating PCI from anterior circulation infarction (ACI) and controls may be present.

**Materials and Methods:** As study candidates, we retrospectively enrolled hospitalized patients with first-time non-AF stroke within 2-years period. Consecutive non-AF, non-stroke subjects were enrolled as the control group. We compared NVAFV values among the PCI, ACI, and control groups.

**Results:** Overall, 866 candidates—213, 418, and 235 candidates in the PCI, ACI, and control groups, respectively—were enrolled. NVAFV (mean ± SD) values were 134.8 ± 52.7, 152.3 ± 59.2, and 172.0 ± 54.7 mL/min in the PCI, ACI, and control groups, respectively. Statistics revealed significant difference (*p* < 0.001) among three groups. To use NVAFV as a diagnostic parameter, the AUC of any two groups should be between 0.58 and 0.69. Most (93.6%) of the controls had NVAFV above 100 mL/min. The odds ratio of any non-AF stroke is 3.48 if the NVAFV is below 100 mL/min.

**Conclusions:** NVAFV is lowest in non-AF PCI group. Low NVAFV is associated with both non-AF ACI and PCI. No ideal cut-off value is available to discriminate PCI from other two conditions. We agree that an NVAFV of 100 mL/min is the lower limit of a normal value. Any value below 100 mL/min indicates high stroke risk and implies diffuse cerebral atherosclerosis and impaired cerebral perfusion.

## Introduction

Net vertebral artery flow volume (NVAFV), calculated from extracranial color-coded duplex sonography (ECCS), is widely used in clinical practice to represent adequacy of posterior circulation. The normal range of NVAFV, defined as the 5th−95th percentile, is between 102.4 and 301.0 mL/min (1). Conventionally, NVAFV above 100 mL/min is considered normal. Early studies demonstrated association between decreased NVAFV and vertebrobasilar insufficiency (2, 3). However, the association between NVAFV and posterior circulation infarction (PCI) and other stroke types is still unclear.

Atrial fibrillation (AF), either in paroxysmal, persistent, or permanent form, is a major concern when we utilize NVAFV to evaluate vessel pathologies. Not only that AF-related embolic occlusion of posterior circulation tends to involve BA and PCA rather than extracranial VA, irregular hemodynamics causes problems of flow volume estimation. AF tends to produce low peak systolic, diastolic, and mean velocities at insonated arteries and hence interferes with NVAFV calculation (4, 5). Considering the true effect of arteriosclerosis on blood flow in PCI, AF can be reasonably managed separately.

In this study, we aimed at analyzing NVAFV in non-AF ACI, PCI, and control groups. We hypothesized NVAFV was low in non-AF PCI patients and an ideal cut-off value for discriminating PCI from ACI and controls may be present. Furthermore, we verified the normal NVAFV of non-stroke controls and calculate odds ratios of any non-AF stroke based on different NVAFV values to help stroke risk stratification in clinical practice.

## Methods

### Study Design and Candidates

The protocol of this study was approved by the Joint Institutional Review Board of Taipei Medical University (N201804065). The board granted a waiver for written informed consent because of the retrospective nature of the study. We reviewed the medical records of all patients hospitalized at the Stroke Center of Taipei Medical University–Shuang Ho Hospital from an established stroke registry system from January 2015 to December 2016. Inclusion criteria of this study was first-time non-AF acute ischemic stroke. Exclusion criteria were simultaneous ACI and PCI, apparently pathologic VA flow and incomplete ECCS data. Three qualified neurologists (H-HL, L-KH, and H-JC) reviewed the brain MRI to ascertain the presence of acute ischemic stroke, involved brain regions, and absence of old brain insults. One senior neurologist (C-CC) confirmed these reviews. For ambiguous cases, consensus was reached through group discussions. Clinical information regarding age, sex, hypertension, dyslipidemia, diabetes, smoking, and TOAST classification of stroke was collected from the registry. Hypertension was defined as systolic blood pressure above 140 mmHg or diastolic blood pressure above 90 mmHg (6). Dyslipidemia was defined as low-density lipoprotein cholesterol levels above 130 mg/dL (7). Diabetes was defined as HbA1c above 6.5% (8). Consecutive non-AF, non-stroke individuals older than 55 years receiving the ECCS study from January to December 2016 as a scheduled health checkup were enrolled as the control group.

### ACI and PCI Criteria

ACI is defined as diffusion-weighted imaging (DWI)-positive infarcts involving the brain territories supplied by the internal carotid, middle cerebral, and anterior cerebral arteries. Typically, infarcts in the frontal, parietal, lateral temporal cortical and subcortical regions, internal capsule, and basal ganglia are recognized as ACI. PCI is defined as DWI-positive infarcts involving the brain territories supplied by the VA, BA, and PCA. Typically, infarcts in the brainstem, cerebellum, thalamus, medial temporal, and occipital regions are recognized as PCI. Patients enrolled in the study group were categorized into either the ACI or PCI group. Patients with uncertain stroke territory or simultaneous ACI and PCI were excluded due to the assumed pathogenesis of cardioembolism or coagulopathy. Patients in the PCI group, excluding those with the isolated distal territory involvement, were further categorized into the brainstem/cerebellum subgroup. We assumed that low VA flow volume may correlate more strongly in this subgroup after the removal of the influence of the isolated distal territory PCI.

### ECCS Estimation of VA Flow Volume

All study patients hospitalized at Stroke Center of Shuang Ho Hospital received a complete extracranial and transcranial duplex evaluation within 1 week after disease onset. The examination procedures were performed using two models of ultrasound machines (Philips IE33 and Philips EPIQ7) by experienced ultrasound technicians. Apparent VA pathological spectral patterns, including trickle, biphasic, reverse, turbulent, and absent diastolic flows, were recorded. Except for the pathological flow, the VA flow volume was calculated from the V2 segment by multiplying TAMV by the cross-area of the sampled segment. NVAFV was obtained through the summation of bilateral VA flow volumes. Patients with unilateral or bilateral pathological VA flow patterns were at a high risk for PCI and hence were not enrolled in this study.

### Statistical Analysis

IBM SPSS Statistics 19 (Windows) was used for statistical computation. Background and risk factors among the ACI, PCI, and control groups and the brainstem/cerebellum subgroup were compared. The parameters included age, sex, hypertension, diabetes, dyslipidemia, and smoking. Age distributions were compared among the groups by using a Student *t*-test. Sex, hypertension, diabetes, dyslipidemia, and smoking were compared among the groups by using a chi squared test. Significant difference was defined as *p* < 0.05. The NVAFV distributions in each group are presented as range, lower quartile, mean, upper quartile, and standard deviation. Frequency plots based on the flow volume at every 20 mL/min increment were created. Visual methods and normality tests were used for assessing normality. Linear regression analysis was used to determine the significance of the confounding factors. The differences in NVAFV among the groups were calculated using parametric or non-parametric analysis. A receiver operating characteristic (ROC) curve was plotted to differentiate any two groups by using NVAFV, and the area under the curve was calculated. We identified an optimal cut-off value of NVAFV and determined its clinical significance and effect by calculating the odds ratio.

## Results

**Figure 1:**
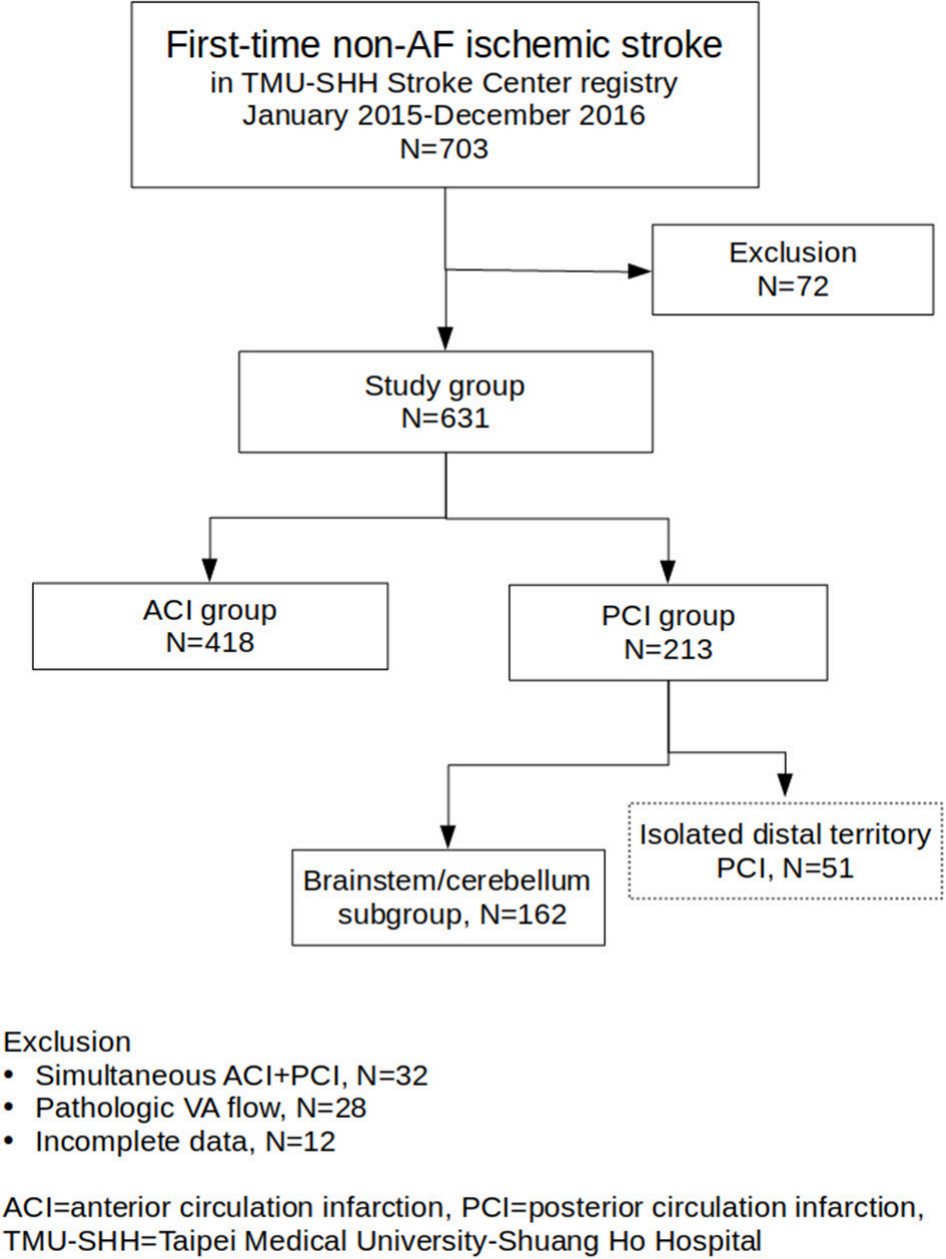
Flowchart of study group enrollment and categorization. From TMU-SHH Stroke Center registry, a total of 631 patients were enrolled. The patients were categorized into ACI group (*N* = 418) and PCI group (*N* = 213). One hundred and sixty two patients in the PCI group were further categorized into the brainstem/cerebellum subgroup.

Successive 703 medical records and brain MRI in the stroke registry system and 240 medical records in the control group were reviewed. In total, 72 subjects from the stroke registry system were excluded because of simultaneous ACI and PCI (32 subjects), apparently pathological VA flow patterns (28 subjects) and incomplete data (12 subjects). Five patients in the control group were excluded because of incomplete clinical information. Among the 631 enrolled stroke patients, 418 (68.2%) and 213 (31.8%) patients were categorized into the ACI and PCI groups, respectively; 162 patients in the PCI group were further categorized into the brainstem/cerebellum subgroup (Figure 1). Demographic information regarding age, sex, diabetes, dyslipidemia, hypertension, smoking, and TOAST classification is summarized in Table 1. Age and sex distributions among the three main groups were similar. Diabetes was more prevalent in the PCI group than in the ACI group (*p* < 0.001). Dyslipidemia was the most prevalent in the control group, but the result was similar between the ACI and PCI groups. The prevalence of hypertension was similar between the ACI and PCI groups. The prevalence of smoking was similar among the three main groups. Small-vessel occlusion was more prevalent than large-artery atherosclerosis in both ACI and PCI groups.

**Table 1:** Demographic information of three main groups and one subgroup.

	**Non-AF ischemic stroke**			**Control group *N* = 235**
	**ACI group *N* = 418**	**PCI group *N* = 213**	**Brainstem/ cerebellum subgroup, *N* = 162**
Age, mean ± SD^†^	66.5 ± 13.7	65.0 ± 13.4	66.1 ± 13.3	63.5 ± 6.1
Male^†^	67.5%	61.0%	59.9%	62.6%
Diabetes^‡^	36.4%	50.7%	54.3%	12.8%
Dyslipidemia^§^	38.5%	34.3%	34.0%	46.8%
Hypertension^¶^	58.4%	65.7%	65.4%	39.6%
Smoking^†^	17.7%	12.2%	12.3%	19.6%
TOAST	Large 29.2%	Large 17.6%	Large 16.2%	N/A
	Small 59.6%	Small 74.1%	Small 75.1%

*AF, atrial fibrillation; ACI, anterior circulation infarction; PCI, posterior circulation infarction; TOAST, Trial of Org 10172 in Acute Stroke Treatment Classification*.

† *No significant difference in prevalence among three main groups*.

‡ *Significant difference in prevalence among three main groups: PCI>ACI>Control*.

§ *Highest prevalence in control group; no significant difference between ACI and PCI groups*.

¶ *Lowest prevalence in control group; no significant difference between ACI and PCI groups*.

**Figure 2:**
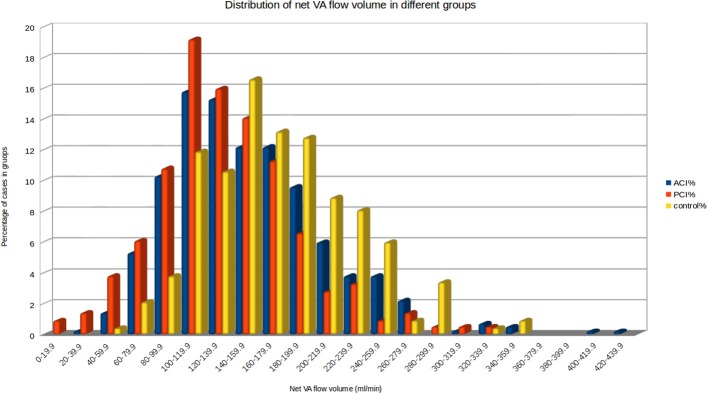
Histogram of net VA flow volume in the three main groups. 93.6% of the patients in the control group had NVAFV above 100 mL/min. By visual inspection, clear cut-off values between any two groups are not obtainable.

NVAFV (mean ± SD, mL/min) were 172.0 ± 54.7 in control group, 152.3 ± 59.2 in ACI group, 134.8 ± 52.7 in PCI group and 132.3 ± 54.4 in brainstem/cerebellum subgroup. The difference between the PCI group and brainstem/cerebellum subgroup was non-significant. Linear regression analysis showed no significant effect of age, sex, and hypertension on NVAFV among three main groups. Mechanisms of stroke by TOAST classification had no significant effect on NVAFV between ACI and PCI groups. Visual methods for accessing NVAFV normality did not show substantial deviation from normal distribution. However, normality test of NVAFV in three main groups revealed the values were not normally distributed (Shapiro-Wilk test, *p* < 0.05). Non-parametric analysis demonstrated a significant difference in NVAFV among three main groups (Kruskal-Wallis test, *p* < 0.001). Pairwise Wilcoxon comparisons confirmed that the differences existed between any two groups (*p* < 0.002). Histogram plots of NVAFV at the 20 mL/min increment in the three main groups are displayed in Figure 2. In the control group, 93.6 and 95.3% of the patients had NVAFV above 100 and 90 mL/min, respectively. The NVAFV was above 100 mL/min for 77% (164/213) and 82.8% (346/418) of PCI and ACI patients, respectively. Overall, 80.8% of non-AF stroke patients bore the same flow volume range as most normal patients. Positive and negative predictive values of stroke with NVAFV below 100 mL/min were 89.0 and 30.1%.

**Figure 3:**
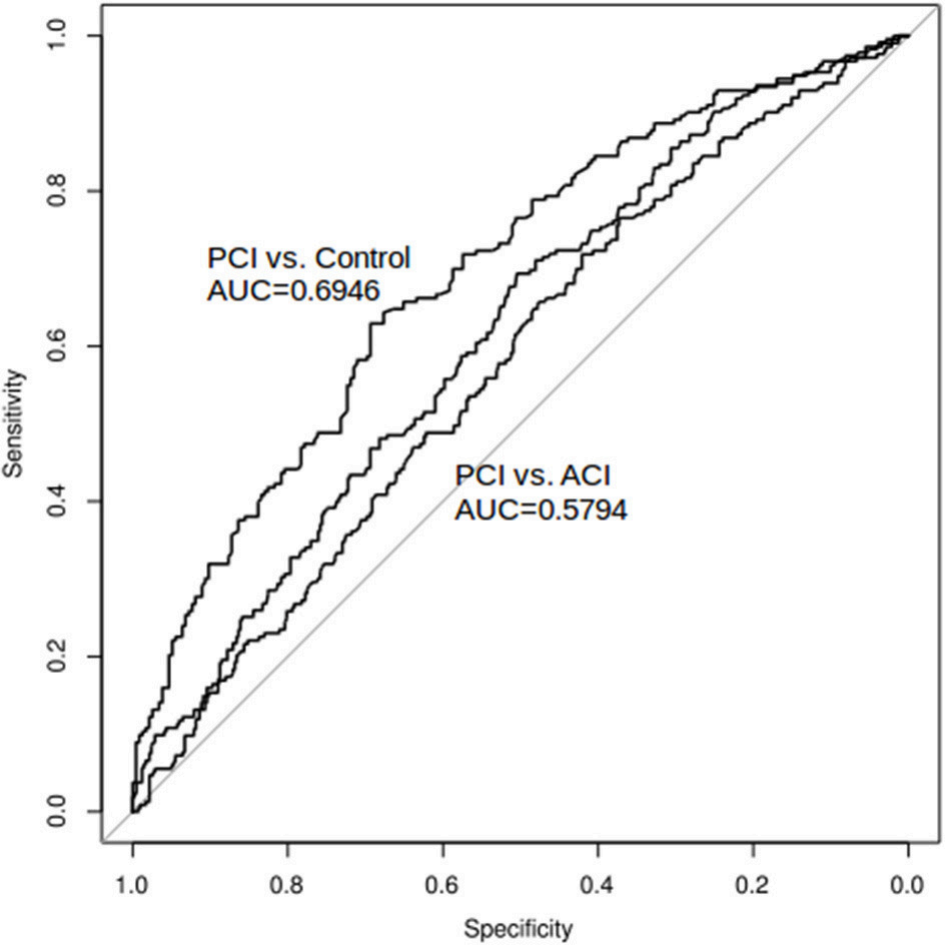
ROC curve and AUC: net VA flow volume as a diagnostic tool to differentiate any two groups. The area under the curve was the largest (0.69) for the PCI than for the control group, with the optimal cut-off threshold at 143.9 mL/min. The area under the curve was the smallest (0.58) for the ACI than for the PCI group.

The ROC curve of NVAFV differentiating any two groups is displayed in Figure 3. The area under the curve was the largest (0.69) for the PCI than for the control group, with the optimal cut-off threshold at 143.9 mL/min (0.69 specificity, 0.63 sensitivity). For determining a clinically applicable cut-off point to differentiate all non-AF stroke patients from the control group, we calculated the odds ratios at different values (Table 2). When the cut-off value was set at 100 mL/min, the odds ratio of any non-AF stroke was 3.48 (1.99–6.09 in 95% CI, *p* < 0.001). When the value was set at 90 mL/min, the odds ratio was 3.26 (1.71–6.21 in 95% CI, *p* < 0.001).

**Table 2:** Odds ratios of non-AF stroke based on various cut-off values of net VA flow volume.

**Cutoff value (mL/min)**	**Non-AF stroke (*N*)**	**Control (*N*)**	**OR (95% CI)**	***p*-value**
<80	55	6	3.64 (1.55–8.58)	<0.002
<90	87	11	3.26 (1.71–6.21)	<0.001
<100	121	15	3.48 (1.99–6.09)	<0.001
<110	176	26	3.11 (2.00–4.84)	<0.001
<120	228	43	2.53 (1.75–3.65)	<0.001

## Discussion

The main findings of this study are as follows: (1) Most (93.6%) of the non-AF, non-stroke controls had an NVAFV above 100 mL/min. (2) The non-AF PCI group had the lowest NVAFV. (3) Low NVAFV is significantly associated with any non-AF stroke. (4) The odds ratios of any non-AF stroke with NVAFV below 90 and 100 mL/min were 3.26 and 3.48, respectively. (5) No safe range of NVAFV exists. (6) Optimal cut-off values of NVAFV to differentiate non-AF stroke patients from control subjects or PCI from ACI are not available.

Atherosclerotic VA diseases most commonly involve the very origin of VA and intracranial VA (9). The criteria for diagnosing >50% stenosis at these segments have been well-documented (10, 11). Therefore, the chance of detecting stenosis or stenotic flow is low while insonating the intersegmental portion (V2) of the VA by using ECCS. Instead, the abnormal flow spectra at V2 are more likely to be post-stenotic or pre-stenotic flow revealing proximal or distal VA disease. Both post-stenotic and pre-stenotic flow patterns reduce the estimated VA flow volume; hence, NVAFV decreases in the state of significant (>50% stenosis) or non-significant (<50% stenosis) VA atherosclerosis.

The finding that NVAFV is the lowest in the PCI group supports the long-held idea that NVAFV helps evaluate posterior circulation adequacy. However, patients did not bear normal NVAFV in the ACI group (relative to the control group). Few discussions have focused on the VA flow volume changes in the condition of carotid artery diseases. Only one study demonstrated a mean increase of 14.29% of NVAFV in patients with unilateral extracranial internal carotid artery occlusion, which is probably due to hemodynamic compensation (12). However, we could not replicate these findings in our ACI group. One possible explanation is that not all patients in our ACI group had severe extracranial carotid disease; hence, compensatory flow from posterior circulation may not necessarily occur. Moreover, we assumed that decreased NVAFV in both the ACI and PCI groups reflects the diffuse cerebral atherosclerosis process, which leads to subsequent ischemic stroke. The effect may be more pronounced in a portion of patients who subsequently had PCI.

Chi et al. (13) used 100 mL/min as a cut-off value to show that 72.5% of stroke patients had normal NVAFV. This finding is close to our result of 80.8%. Although low NVAFV was more prevalent in the PCI group, no significant difference between ACI and PCI groups was observed (*p* = 0.25). We agree that NVAFV fails to precisely differentiate PCI from ACI, but the value of this parameter may be underrated. After excluding AF-related stroke patients and including control individuals for comparison in our study, the NVAFV parameter proved to be a valuable supplement to a seemingly pathological VA flow pattern and probably reflects the degree of cerebral atherosclerosis. NVAFV is a favorable index that could be incorporated into the current criteria of the ABCD3-I score to stratify the risk of stroke (14).

Some limitations of this study are addressed herein. Firstly, the influence of unilateral or bilateral hypoplasic VA on NVAFV was not analyzed. Because of the current lack of consensus on the definition of VA hypoplasia, we did not include this as a confounding factor in our study. Secondly, in some cases, the ACI and PCI judgment could be arbitrary. For example, problems may arise in the face of a fresh watershed infarct between MCA and PCA territory, along with a unilateral full fetal-type PCA with a fresh ipsilateral occipital infarct. As is protocol, we did not refer to the angiographic data; therefore, in a few cases, the categorization may not exactly match the true mechanism. Thirdly, all AF patients were excluded from this study because we assumed that AF causes embolic stroke and AF-related stroke does not display VA hemodynamic abnormalities. This assumption may not be true for all AF patients because an AF patient with a stroke mechanism of atherosclerosis is not uncommon. Finally, we could not confirm whether low NVAFV was the cause or the result of stroke. To our knowledge, studies clearly demonstrating influence of acute stroke on ECCS flow volume change are still lacking.

## Conclusion

The use of NVAFV as a diagnostic parameter is compromised by its lack of accuracy. We could not identify a suitable cut-off value for clinicians to differentiate patients with PCI from those with ACI or those with PCI from normal individuals. The conventionally used cut-off value of 100 mL/min is suitable as a lower limit for normal individuals, but it does not necessarily predict or rule out posterior circulation ischemia. Nevertheless, we advocate the use of this parameter as a screening tool to evaluate the degree of cerebral atherosclerosis and to stratify the risk of non-AF stroke. Per this study, the odds ratios for any non-AF stroke is 3.48 and 3.26 with NVAFV below 100 and 90 mL/min, respectively.

## Ethics Statement

The study was approved by the local ethics committee and was performed in accordance with the ethical standards of the 1964 Declaration of Helsinki.

## Author Contributions

H-HL and L-KH drafted the manuscript. H-JC, DW, N-FC, and LC collected candidate information. C-JH and C-CC analyzed datasets and edited the manuscript.

### Conflict of Interest Statement

The authors declare that the research was conducted in the absence of any commercial or financial relationships that could be construed as a potential conflict of interest.

## Abbreviations

ACI: anterior circulation infarction
AF: atrial fibrillation
BA: basilar artery
ECCS: extracranial color-coded duplex sonography
DWI: diffusion-weighted image
NVAFV: net vertebral artery flow volume
PCA: posterior cerebral artery
PCI: posterior circulation infarction
VA: vertebral artery
TAMV: time-averaged mean velocity
TIA: transient ischemic attack.
